# Alteration of Proteins and Pigments Influence the Function of Photosystem I under Iron Deficiency from *Chlamydomonas reinhardtii*

**DOI:** 10.1371/journal.pone.0035084

**Published:** 2012-04-13

**Authors:** Venkateswarlu Yadavalli, Craig C. Jolley, Chandramouli Malleda, Balakumar Thangaraj, Petra Fromme, Rajagopal Subramanyam

**Affiliations:** 1 Department of Biochemistry, School of Life Sciences, University of Hyderabad, Hyderabad, India; 2 Department of Chemistry and Biochemistry, Center for Bioenergy and Photosynthesis, Arizona State University, Tempe, Arizona, United States of America; 3 Department of Plant Sciences, School of Life Sciences, University of Hyderabad, Hyderabad, India; United States Department of Agriculture, Agricultural Research Service, United States of America

## Abstract

**Background:**

Iron is an essential micronutrient for all organisms because it is a component of enzyme cofactors that catalyze redox reactions in fundamental metabolic processes. Even though iron is abundant on earth, it is often present in the insoluble ferric [Fe (III)] state, leaving many surface environments Fe-limited. The haploid green alga *Chlamydomonas reinhardtii* is used as a model organism for studying eukaryotic photosynthesis. This study explores structural and functional changes in PSI-LHCI supercomplexes under Fe deficiency as the eukaryotic photosynthetic apparatus adapts to Fe deficiency.

**Results:**

77K emission spectra and sucrose density gradient data show that PSI and LHCI subunits are affected under iron deficiency conditions. The visible circular dichroism (CD) spectra associated with strongly-coupled chlorophyll dimers increases in intensity. The change in CD signals of pigments originates from the modification of interactions between pigment molecules. Evidence from sucrose gradients and non-denaturing (green) gels indicates that PSI-LHCI levels were reduced after cells were grown for 72 h in Fe-deficient medium. Ultrafast fluorescence spectroscopy suggests that red-shifted pigments in the PSI-LHCI antenna were lost during Fe stress. Further, denaturing gel electrophoresis and immunoblot analysis reveals that levels of the PSI subunits PsaC and PsaD decreased, while PsaE was completely absent after Fe stress. The light harvesting complexes were also susceptible to iron deficiency, with Lhca1 and Lhca9 showing the most dramatic decreases. These changes in the number and composition of PSI-LHCI supercomplexes may be caused by reactive oxygen species, which increase under Fe deficiency conditions.

**Conclusions:**

Fe deficiency induces rapid reduction of the levels of photosynthetic pigments due to a decrease in chlorophyll synthesis. Chlorophyll is important not only as a light-harvesting pigment, but also has a structural role, particularly in the pigment-rich LHCI subunits. The reduced level of chlorophyll molecules inhibits the formation of large PSI-LHCI supercomplexes, further decreasing the photosynthetic efficiency.

## Introduction

Iron in aerobic soils and waters is found mostly in the form of insoluble Fe(III) oxides, while Fe(II) is typically not found in high concentrations in the environment under aerobic conditions [1]. There is now strong evidence that Fe limitation controls algal productivity in large parts of the open ocean [2], [3]. Organisms have developed complex systems for Fe acquisition, and for adjusting their biochemical pathways to survive in low Fe environments. The majority of Fe in algae and plants is believed to be associated with the chloroplast [4]. In oxygenic photosynthesis, Fe is a cofactor in photosystem (PS) II, PSI, the cytochrome (Cyt) *b*_6_/*f* complex, and (in algae) Cyt *c_6_*. The abundance of these proteins decreases during Fe deficient growth [5].

On the molecular level, PSI is a prime target of Fe deficiency because of its high Fe content (12 Fe per PSI). The ratio of PSI/PSII changes from 4∶1 to 1∶1 under Fe deficiency in cyanobacteria [6] and there is a diatom that adapts to low ambient Fe by changing its PSII/PSI ratio constitutively to about 10∶1 [7]. The mechanistic details of Fe stress response differ among phototrophs, but the various approaches seek to address similar physiological demands. Because of PSI's high Fe requirements, Fe deprivation leads to a decrease in the number of PSI complexes, resulting in a bottleneck in the photosynthetic electron flow [7]. To avoid oxidative stress, many species express auxiliary light-harvesting proteins which increase the absorption cross-section of PSI, allowing the cell to maintain a similar level of electron throughput with a smaller Fe investment [8].

In higher plants, Fe deficiency leads to all of these effects: a decrease in the abundance of photosynthetic proteins [9], reduction of electron transport chain components [10], a decrease in PSI levels [11], and a decrease in the quantum yield of PSII [12]. Cyanobacteria respond to Fe deficiency by expression of the “iron-stress-induced” operon *isiAB* [13]. Recently, Chauhan *et al.*, [8] showed that PSI-IsiA supercomplexes with two concentric layers of IsiA around PSI form during prolonged growth at nanomolar Fe levels.

In the obligate photoautotrophic green alga *Dunaliella salina*, a chlorophyll *a*/*b*-binding protein homolog is induced by Fe deficiency and functionally associated with PSI [14]. Fe deprivation in *D. salina* triggers extensive changes in chloroplast morphology, photosynthetic activities, and the expression of a major 45 kDa chloroplast protein termed Tidi which resembles the chlorophyll *a*/*b*-binding proteins present in light-harvesting antenna complexes (LHC) [14]. Single particle electron microscopic analysis revealed that PSI particles from Fe-stressed *D. salina* cells are larger (31 and 37 nm in diameter) than complexes from control cells (22 nm) [14]. The 77 K chlorophyll fluorescence emission spectra of isolated complexes suggest that the Tidi-LHCI antenna system is functionally coupled to the reaction center of PSI. These findings indicate that Tidi acts as an accessory antenna of PSI, similar to IsiA in cyanobacteria.

In the green alga *C. reinhardtii*, the initial stages of Fe stress involve not only reduced levels of PSI, but also a remodeling of the PSI-associated LHCI, resulting in decreased amount of PSI, and decreased PSI antenna size [15]–[17], and increase of expression of protein complexes in respiration [16]. Furthermore Chlamydomonas cells grown under Fe-deficiency are very sensitive to high light stress and oxidative damage and a proteomics study has shown that enzymes involve in oxidative stress response are strongly overexpressed under iron deficiency [16]. The decrease in overall photosynthetic efficiency and decrease of total amount of PSI per cell is observed both in cyanobacteria and green algae however the structural and functional changes in the function of electron transport and excitation energy transfer in PSI have not been studied in detail in green algae. Naumann and coworkers reported that Fe deficiency causes a decrease in ratio of LHCI proteins to PSI in green algae [15]. The question why green algae decrease the size of the PSI antenna (instead of increasing the efficiency of light harvesting as found in cyanobacteria) has not yet been answered.

We have used *C. reinhardtii* as a model system to study in more detail the specific effects of Fe stress conditions on PSI-LHCI supercomplexes. The present study focuses on changes in the pigment-pigment interactions, the protein profile of the PSI core and LHCI complexes, and changes in the excitation energy transfer processes in PSI-LHCI supercomplexes.

## Methods

### Isolation of PSI-LHCI supercomplexes

*C. reinhardtii* cells (obtained from the *Chlamydomonas* culture collection at Duke University) were grown following standard procedures [18], [19]. An initial culture of *C. reinhardtii* cells was harvested and washed twice with Fe-free tris acetate phosphate (TAP) media and then inoculated into a 1.0 L culture flask. Cells were grown under both control and Fe deficiency conditions with continuous illumination (30–40 µmol m^−2^ s^−1^). Cells were then lysed and thylakoid membranes isolated according to Fischer et al. [20], and resuspended in 200 mM sorbitol, 5 mM Tris–HCl (pH 8.0), 5 mM CaCl_2_. PSI-LHCI supercomplexes were isolated as previously described [18], [21], [22]. Sucrose density gradients were prepared by stepwise addition of different concentrations of sucrose (2M, 1M, 0.75M, 0.5M, and 0.25M from bottom to top) containing 5 mM Tricine pH 8.0 and 0.5M betaine. Following sucrose density gradient centrifugation, the lowest band, containing PSI-LHCI supercomplexes [18], [19], was collected and diluted with three volumes of 5 mM Tris–HCl pH 8.0, 0.05% β-dodecyl maltoside (DDM), 5 mM CaCl_2_. The diluted samples were concentrated using a Centricon-100 ultrafiltration device (Amicon, Beverly, MA, USA) by centrifuging at 4,000×*g* in a Sorvall SS-34 rotor at 4°C. To observe temporal changes in PSI-LHCI supercomplexes under Fe-deficient growth conditions, cells were harvested at 24, 48, and 72 h. Unless indicated otherwise, the experiments described below utilized samples isolated from cells subjected to 72 h of Fe limitation. For the assays described below, all samples were diluted to equal protein or Chl concentrations as needed. Control (*i.e.* non-stressed) samples came from cells grown in TAP medium for 72 h.

### Superoxide dismutase (SOD) activity assay

For SOD activity measurements, 50 µg of thylakoid membranes were resuspended in 10 mM Tris–HCl (pH 7.5), 0.3 M sorbitol, 1.5 mM CaCl_2_, 10 mM MgCl_2_. The in-gel SOD assay and activity measurements were performed according to Beauchamp and Fridovich [23]. Thylakoids and SOD reaction buffer were mixed in an Eppendorf tube and illuminated for 12 min using a Comptalux bulb. Absorbance was measured at 560 nm after cooling the sample to room temperature.

### Low-temperature emission fluorescence measurement

Steady-state fluorescence emission spectra of intact cells at 77K were measured with a LS-55 fluorescence spectrophotometer (PerkinElmer, USA). Cells were resuspended in growth medium containing 60% glycerol. Excitation was set at 436 nm and the emission range was from 650–750 nm [24]. The emission and excitation slit width were set at 5 nm.

### Circular dichroism (CD) measurements

Visible circular dichroism spectra were measured in a J-810 spectropolarimeter (Jasco Inc., Easton, MD, USA) [22]. The optical path length of the cell was 1 cm, and the distance of the sample from the photomultiplier was 5 cm. The spectra were recorded in 1 nm steps with an integration time of 0.3 s and a band pass of 2 nm. Equal Chl (10 µg/mL) concentration was used for measuring the CD.

### Green gel electrophoresis

PSI-LHCI supercomplexes were solubilized in 50 mM Tricine, 5 mM EDTA pH 7.5, 50% Glycerol, and 0.5% DDM. The protein composition of the sample was resolved on 4–15% polyacrylamide gel electrophoresis (PAGE) gels with 25 mM Tris, 250 mM Glycine, 0.005% Lithium dodecyl sulphate running buffer containing 0.05% Deriphat at 4°C [25].

### SDS-denaturing gel electrophoresis

For the analysis of the protein composition of *C. reinhardtii* PSI-LHCI supercomplexes under control and Fe deficiency conditions, samples were size-fractionated by gradient SDS-PAGE (15–23%) as described in Subramanyam et al. [22]. Equal amounts of protein (100 µg) were loaded on each lane. The PSI-LHCI supercomplexes were solubilized in 2% SDS and 0.1 M dithiothreitol before loading the sample. The gel was stained with Coomassie Brilliant Blue R250.

### Immunoblot analysis

PSI-LHCI polypeptides were separated on SDS-PAGE. Electrophoresis was performed on a 12% separating and 4% stacking gel of polyacrylamide. Equal quantities of protein were loaded onto each lane. An equal volume of 2× sample buffer was added to the aliquots. To identify and quantify the polypeptides contained in the PSI-LHCI supercomplex, immunoblotting was carried out essentially as described by Towbin et al. [26]. Immunoblotting was performed by electrophoretic transfer of proteins to PVDF membranes. The membrane was incubated with polyclonal antibodies developed in rabbits. Primary antibodies against PsaA, PsaB, PsaC, PsaD, and PsaE were purchased from Agrisera (Vännäs, Sweden). Peptide tag antibodies for Lhca complexes were developed in our laboratory. Subsequently, secondary antibodies ligated to horseradish peroxidase were applied. Chemiluminescence reagents were used to develop the signal on the PVDF membrane. The images were recorded on a VersaDoc 5000 CCD camera (Bio-Rad).

### Ultrafast fluorescence spectroscopy

The time versus wavelength fluorescence intensity surfaces were recorded on a system consisting of an ultrafast laser and a streak camera, as described previously [8], [27]. Briefly, 130 fs light pulses at 800 nm were generated by a mode-locked Ti∶S laser (Mira 900, Coherent Laser Inc., Santa Clara, CA, USA) pumped by a frequency-doubled Nd∶YVO_4_ laser (44% from an 18 W Verdi, Coherent Laser Inc.). The repetition rate of the Ti∶S laser was reduced to 4.75 MHz by a pulse picker (Model 9200, Coherent Laser Inc.). The excitation light (800 nm) was frequency doubled to 400 nm and focused onto a sample cuvette with a 5 mm path length. Fluorescence was collected at a right angle to the excitation beam and focused on the entrance slit of a Chromex 250IS spectrograph, which was coupled to a Hamamatsu C5680 streak camera with a M5675 synchroscan sweep unit. The streak images were recorded on a Hamamatsu C4742 CCD camera. Measurements were performed on 800 ps, 1.4, and 2 ns timescales, with 1,024 pixels of time resolution. The FWHM of the overall time response of this system was ∼6 ps at the 800 ps timescale, ∼12 ps at the 1.4 ns timescale, and ∼20 ps at the 2 ns timescale. The spectral resolution was 0.124 nm in the spectral range of 650–777 nm (1,024 pixels). To avoid singlet–singlet annihilation, the pulse energy was reduced to 0.1 nJ using a neutral density filter. The probability of a single supercomplex absorbing multiple photons in a single laser pulse was estimated using Poisson statistics [28] and it was found that less than 2% of emitted photons could have come from supercomplexes which had received multiple excitations. Global analysis was performed using locally written software in MATLAB. The 1,024 kinetic traces were binned, resulting in a spectral resolution of 1.24 nm. A Gaussian shaped instrument response function was used in the fitting.

## Results

### Analysis of Chlorophyll content

**Figure 1:**
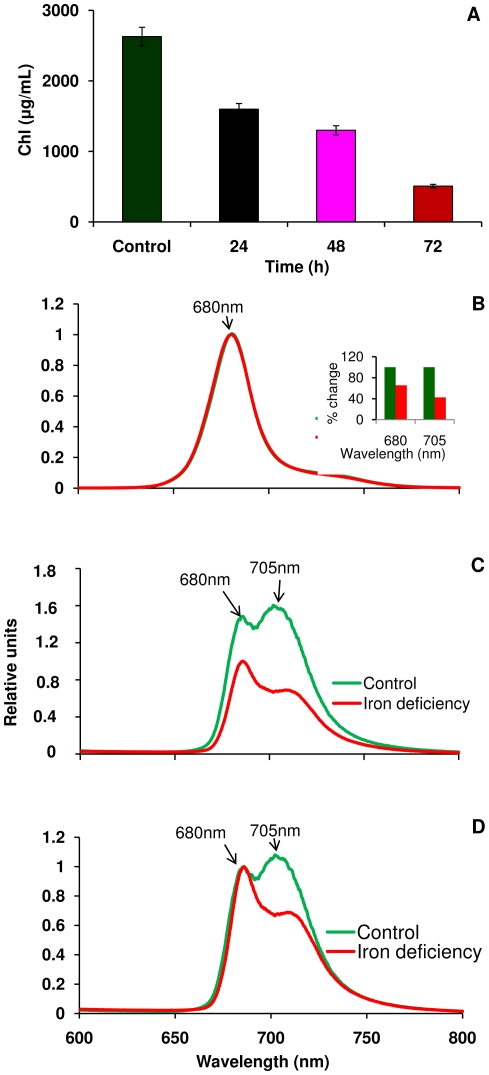
Chlorophyll analysis and fluorescence emission spectra of *C. reinhardtii* cells. A) Chlorophyll content, B) room temperature emission spectra, C) 77K spectra emission spectra, (insert, quantification of the PSI and PSII based on the Figure 1B), and D) 77K emission spectra normalized at 680 nm.

Under Fe deficiency conditions, cells showed a decrease in Chl content over time (Figure 1A). The Chl content decreased by 40% after 24 h, 50% after 48 h, and 80% after 72 h under Fe deficient conditions relative to the control. The cell growth rate also decreased under Fe deficiency conditions relative to the control culture, as determined by the wet weight of the cell pellet.

### Analysis of 77K fluorescence emission

Figure 1B to D shows the steady state room temperature fluorescence emission data of *C. reinhardtii* cells grown under control and Fe limited conditions. Room temperature fluorescence spectra (Figure 1B) show very little difference in the fluorescence maxima at 680 nm between control and Fe-deprived cells, which are measured at equal Chl (2 µg) concentrations. The 77 K emission spectrum is dominated by a small number of red-shifted chlorophyll sites, and is a more sensitive monitor of changes in both PSI and PSII. The 77K emission spectrum reveals fluorescence maxima at 680 and 705 nm which are attributed to PSII and PSI respectively [29]. The amplitudes of these two peaks change dramatically under Fe stress; this can be seen in Figure 1C, and the changes in their relative amplitudes are more conspicuous when the spectra are normalized at 680 nm (Figure 1D). These data indicate that both PSII and PSI were affected, however PSI is more affected than PSII after 72 h of cell growth under Fe deficiency. The per cent change of PSI and PSII fluorescence between control and Fe deficient cells was shown as bar graphs in the insert of Figure 1C.

### Sucrose gradient analysis of PSI-LHCI supercomplexes

**Figure 2:**
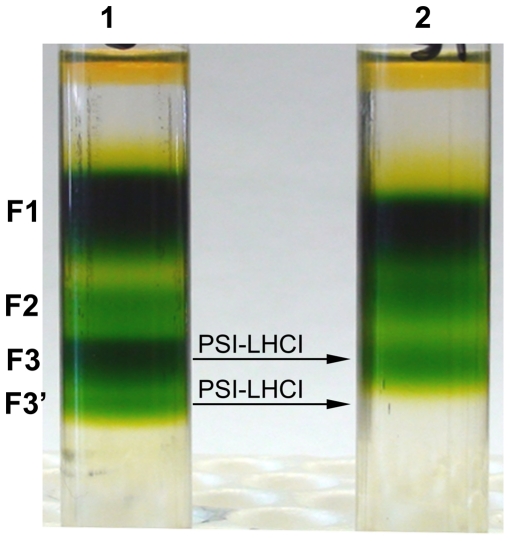
Fractions of PSI-LHCI supercomplexes from solubilized thylakoid membranes by sucrose density centrifugation. F1 (LHCII), F2 (PSI-LHCI and PSII), F3 and F3' (Both contains PSI-LHCI supercomplexes).

PSI-LHCI supercomplexes were isolated using sucrose density gradient centrifugation. The detergent extracts of the thylakoid membranes were loaded onto the gradient at equal chlorophyll levels (0.8 mg/mL Chl). After the centrifugation, the pattern of various green bands was found to differ between the control and Fe-deficient conditions (Figure 2). The control sample contained fractions which are designated F1 (LHCII), F2 (a small PSI-LHCI complex containing 4 LHCI proteins, co-purifying with PSII), F3, and F3' (both containing larger PSI-LHCI supercomplexes with 10–12 LHCI/PSI core), as detailed in a recent study [18]. For this study, PSI-LHCI supercomplexes (F3 for both the control and stress samples, and F3' for the control) were collected from the sucrose gradient. The intensity of the F3 band was diminished in Fe stress conditions, while the F3' band was completely absent (Figure 2), implying that PSI-LHCI supercomplexes are more sensitive to Fe deficiency than other Chl-binding thylakoid proteins.

### Visible CD analysis

**Figure 3:**
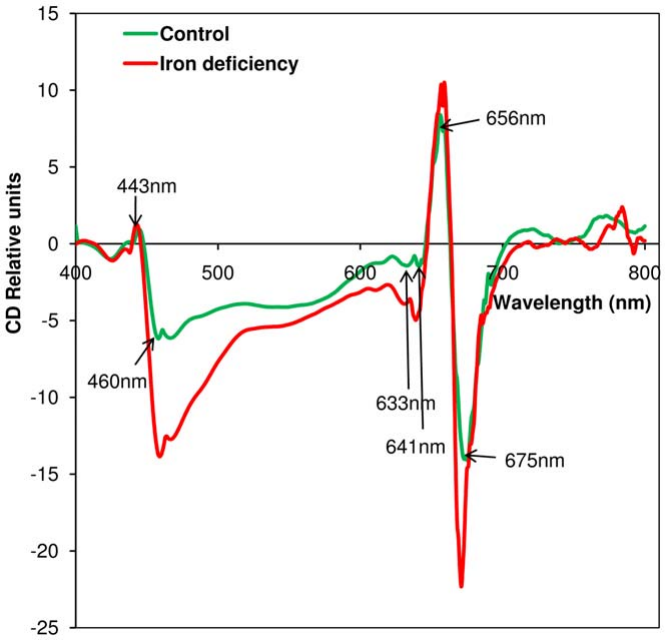
Visible CD spectra of isolated PSI–LHCI supercomplexes from control and Fe stress *C. reinhardtii* cells. The Chl content of the samples was adjusted to 10 µg/mL. CD was measured in absorbance units and for easy comparison it was normalized the spectra at 750 nm.

Visible CD spectroscopy is a sensitive technique to monitor excitonic pigment–pigment and pigment–protein interactions. In the Q_y_ region, the CD spectrum of the isolated PSI-LHCI supercomplexes shows two negative peaks at 641 and 675 nm and one positive peak at 656 nm (Figure 3), similar to previously reported spectra [18], [22]. The two major bands at 656 and 675 nm are due to excitonically-coupled Chl *a* dimers in PSI–LHCI supercomplexes, while the negative peak at 641 nm is characteristic of Chl *b* [18], [22], [30]. In the soret region, the positive peak at 443 nm originates from Chl *a*, while the negative peak at 460 nm is characteristic of Chl *b*. The visible CD spectrum of PSI-LHCI supercomplexes isolated from Fe-stress cells shows an increase in the amplitude of the major peak at 675 nm and a blue shift of about 5 nm. The negative peak at 641 nm showed an increase in amplitude relative to the control, and had a blue shift of about 4 nm. The soret band peaks at 443 and 460 nm also increased in intensity (Figure 3). The differences in the peak intensity indicate changes in excitonic pigment interactions in PSI-LHCI complexes, originating from modification of the spatial relationships between pigment molecules.

### Pigment complex analysis

**Figure 4:**
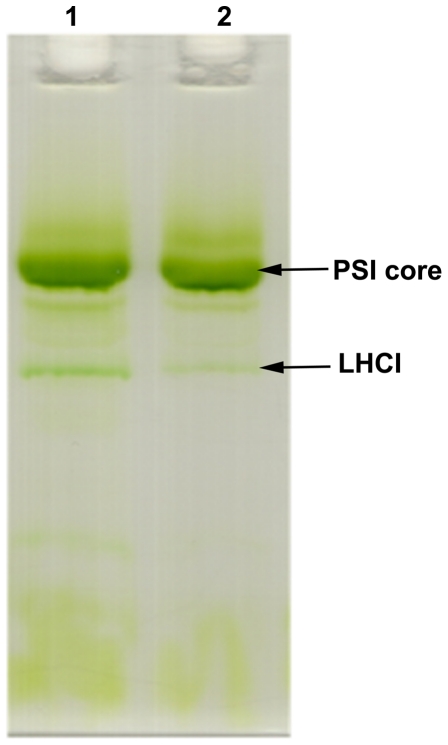
Green gel electrophoresis of PSI-LHCI supercomplexes. Lane 1- Control PSI-LHCI supercomplexes, and Lane 2-PSI-LHCI supercomplexes isolated from Fe deficient culture.

Green gel electrophoresis was performed in order to separate the different pigment-protein complexes of PSI and its associated LHCI proteins. Figure 4 shows a clear separation of pigment complexes of PSI core and LHCI complexes. The results indicate that the number of LHCI proteins decreases under iron deficiency, *i.e.*, the LHCI/PSI ratio in the complexes decreases relative to the control. This indicates that larger PSI-LHCI complexes were not formed in Fe-stressed cells.

### Streak camera data analysis

Fluorescence decay spectra were measured using an ultrafast streak camera setup for PSI-LHCI supercomplexes isolated under Fe stress and control conditions. For each sample, kinetics were measured on 800 ps, 1.4, and 2 ns timescales and fit to three decay components. The component with the shortest lifetime was very consistent across samples and timescales, with a lifetime of ∼33 ps. This is consistent with the behaviour previously observed for *C. reinhardtii* PSI-LHCI under salt stress conditions [22]. Based on comparison with previous studies of excitation trapping in PSI [31], this component of the fluorescence decay can be attributed to the trapping of excitons originating within the PSI core. The second component, which has previously been associated with the trapping of excitons originating in the peripheral LHCI complexes [32], is more heterogeneous. As is shown in Table 1, however, the variation between samples measured at different time scales is more significant than the differences between the three samples. Although fitting of the fluorescence decay with three exponentials provides a convenient description of the kinetics, it must be kept in mind that the real process being measured is far more complicated; the lifetimes found by global analysis merely provide characteristic time scales for various decay processes. Variation of the lifetime found by global analysis with measurement timescale can be attributed to heterogeneity within this decay phase.

**Table 1:** Trapping lifetimes obtained from streak camera measurements.

Sample	*τ*_1_	*τ*_2_
Control	32±4 ps	172±42
Fe stress	34±1.5 ps	165±6
**Timescale**		
800 ps	30±3.5 ps	136.±24
1.4 ns	33±2.5 ps	161±13
2 ns	35±2 ps	196±21

**Figure 5:**
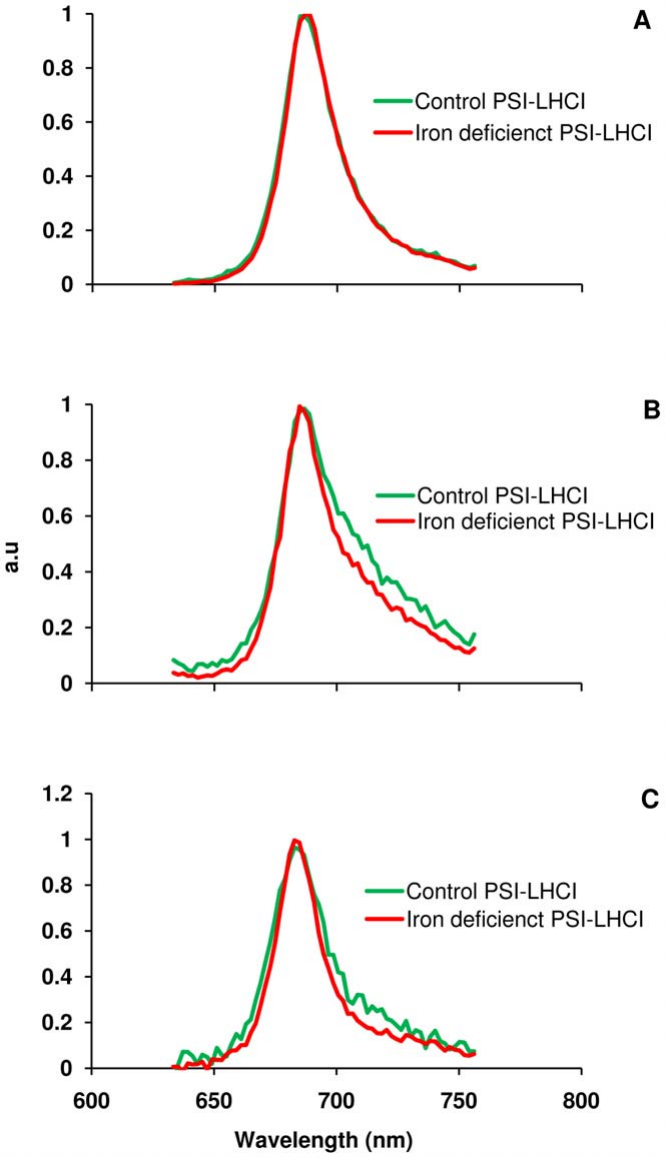
Representative fluorescence decay-associated spectra (FDAS) obtained from fluorescence streak camera measurements from isolated PSI-LHCI supercomplexes of *C. reinhardtii* grown under control and Fe deficient conditions. Fluorescence decay lifetimes are 32–34 ps (A), 120–220 ps (B), and 3–4 ns (C). Each curve shown is the average of the fitted curves from the three data sets collected for each sample.

Only the lifetimes of the first two components are presented; the third was typically fixed at 4 ns during fitting and does not represent functional antenna pigments. These most likely correspond to pigments which are not functionally coupled to the Chl network in PSI or the LHCI proteins. Values are mean ± SE (*n* = 8). Far more insight into the nature of these decay phases can be obtained by examining the spectral shapes of the three decay components. The shape of the first (∼33 ps) component is extremely consistent among the samples measured (Figure 5A). The PsaA and PsaB proteins, which bind the bulk of PSI's core antenna, are largely unaffected by Fe stress, as shown by immunoblotting results. This kinetic phase is likewise unaltered by Fe stress, suggesting that only pigments bound to the PSI core contribute to this component. This is consistent with structural studies [33], [34], which show that the PSI core is a stable, tightly integrated complex. Similar conservation can be seen in the spectral shapes of the third component (3–4 ns); such lifetimes are typically attributed to detergent-solubilized Chl that is not coupled to PSI (Figure 5C). It should be noted that the amplitude of the nanosecond component was much lower in the control sample than in the stress samples and the spectral shapes could only be compared after normalization. This may be a result of decreased stability of the PSI-LHCI complexes from stressed cells relative to the control, resulting in the uncoupling of pigments during purification.

The major difference in the decay-associated spectra of the control and Fe-stress samples can be seen in the spectral shape of the 120–220 ps component (Figure 5B). While the decay lifetimes are relatively unchanged by cell growth under Fe stress conditions, this component shows a decrease in emission intensity on the red side of the peak in the Fe stress samples. Red emission in *C. reinhardtii* PSI has previously been associated with strongly coupled pigments bound by the LHCI proteins [35]–[37] and so this loss of red emission can be attributed to a change in the LHCI antenna system. Similar results were observed in salt stress conditions, suggesting that LHCI is liable to be lost under a variety of different stress conditions [22].

### Immunoblot analysis of PSI core subunits

**Figure 6:**
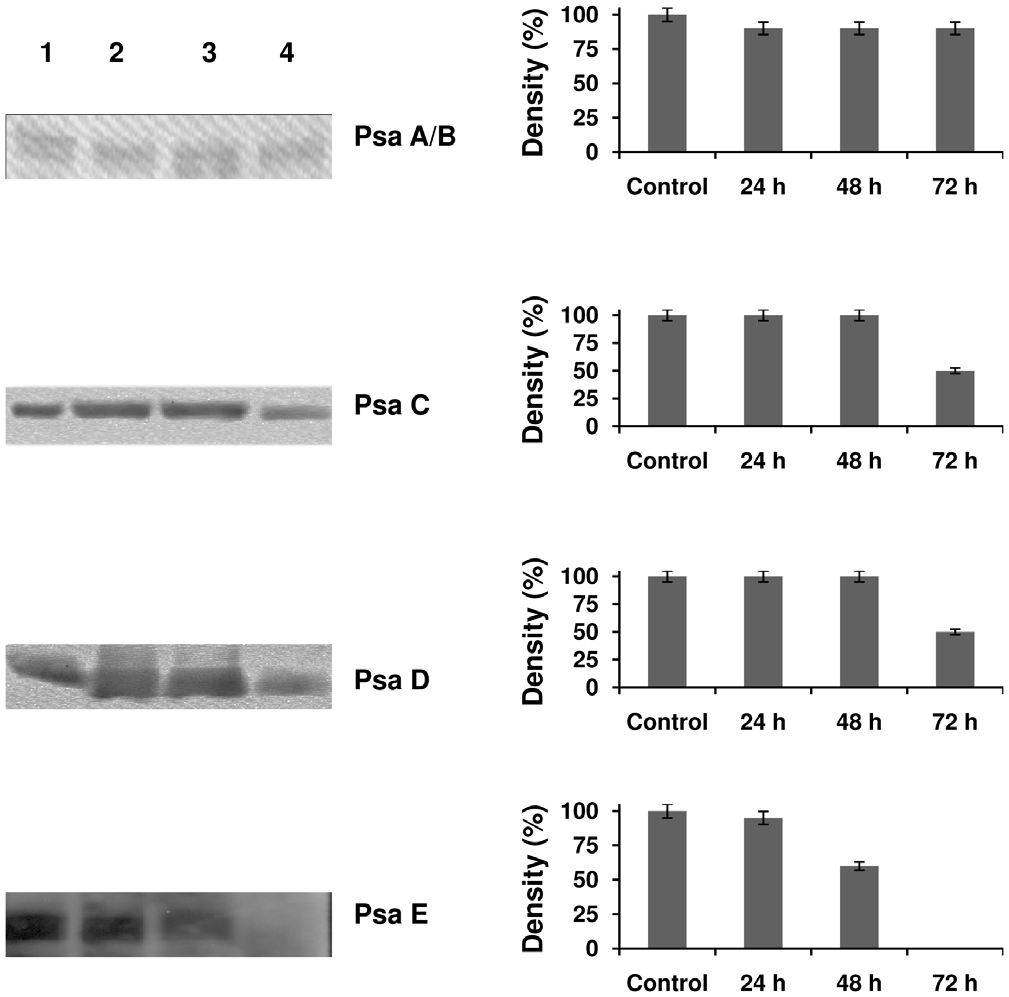
Immuno blot of PSI core subunits were identified from isolated PSI-LHCI supercomplexes. PsaA, PsaB were stable under Fe deficiency conditions while PsaC, PsaD and PsaE content decreased under Fe deficiency. PsaC and PsaD are decreased after 72 hours of Fe deficiency, while PsaE is completely absent in the Fe deficient sample after 72 hours. Lane 1- control, lane 2- 24 h under Fe deficiency, lane 3- 48 h under Fe deficiency, and lane 4- 72 h under Fe deficiency. The bar diagram represents the quantity of the respective protein measured using quantity one 1D analysis software (Biorad).

The proteins were separated by SDS-gel electrophoresis. Immunoblotting was carried out to determine the changes in protein levels in both PSI and LHCI. There was no significant decrease in the core proteins *viz.*, PsaA, PsaB, PsaC, PsaD, and PsaE after 24 h of Fe deficiency. Significant differences in protein composition begin to appear between 48 and 72 h of Fe deficiency. The levels of PsaC and PsaD decreased in the 72 h sample, while the PsaE content decreased after only 48 hours, and was completely absent after 72 h of Fe deficiency (Figure 6). The result that serious decay of the stromal subunits in *C. reinhardtii* under iron deficiency occurs in just 3 days, while the core subunits PsaA and PsaB are still unaffected is astonishing. The same order of decay (loss of PsaE followed by PsaC and PsaD) is observed in cyanobacteria [8] when the cells are grown under complete iron deprivation for more than 3 weeks, at this point the cells also start to show severe chlorosis. However, cyanobacteria can survive and grow for months at nanomolar iron concentration. Under these conditions, they reduce the total amount of PSI per cell but compensate for the reduced amount of PSI by forming the PSI-IsiA double ring structure which increases the light harvesting antenna/P700 and electron flux through individual PSI complexes by factor of 3. Under these conditions the electron transfer chain in PSI is intact and no decay of subunits of the stromal hump, consisting of PsaC, PsaD and PsaE is observed [8].

### Immunoblot analysis of LHCI subunits

**Figure 7:**
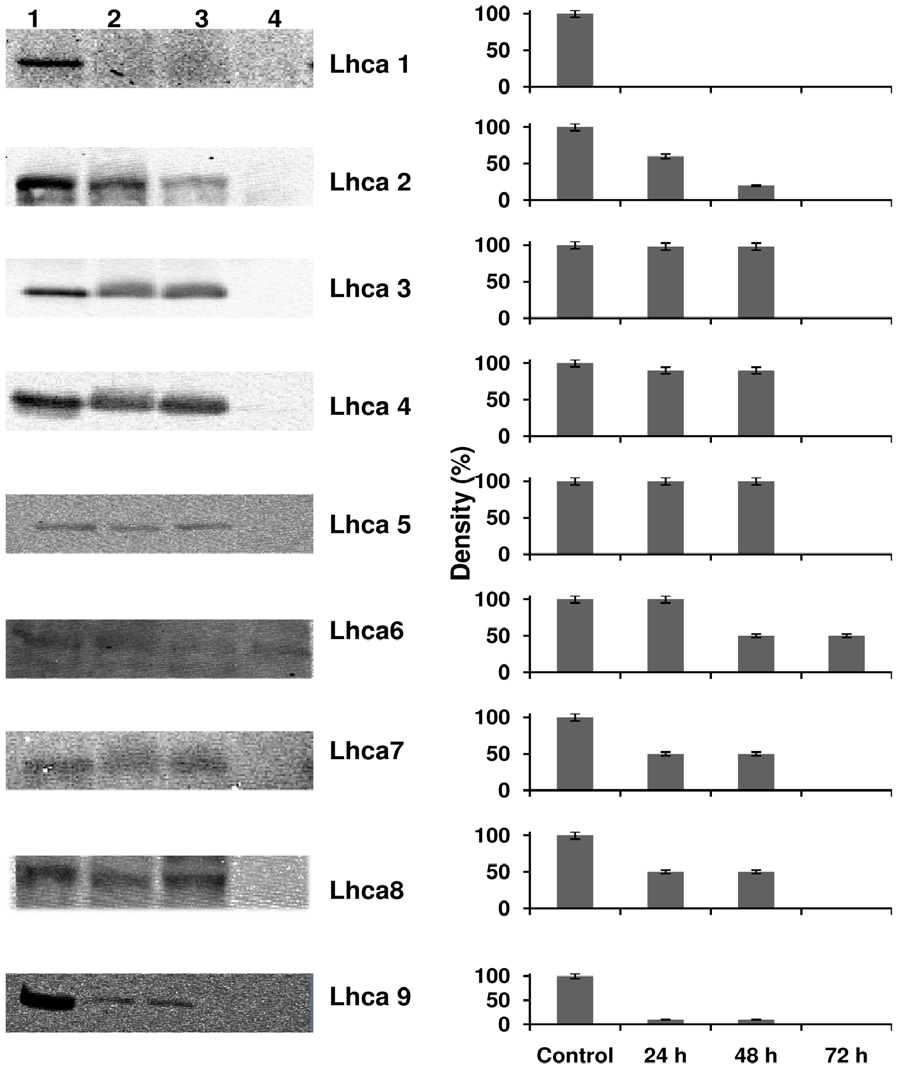
Immuno blot of Lhca 1–9 polypeptides were identified from isolated PSI-LHCI supercomplexes. Lhca 1 and Lhca 9 were more susceptible to Fe deficiency conditions. Lhca 6 was more stable compare to all other LHCI subunits. Lane 1- control, lane 2- 24 h under Fe deficiency, lane 3- 48 h under Fe deficiency, and lane 4- 72 h under Fe deficiency. The bar diagram represents the quantity of the respective protein measured using quantity one 1D analysis software (Biorad).

The LHCI subunits show varying susceptibility to Fe deficiency conditions (Figure 7). After 24 h of Fe deficiency conditions, Lhca1 was found to be absent from the PSI-LHCI supercomplexes, while about 80% of Lhca9 was lost. Lhca2, Lhca7, and Lhca8 were depleted by 50%, while Lhca3, Lhca4, Lhca5, and Lhca6 remained almost unchanged. After 48 h of Fe stress, Lhca2 was depleted to a greater degree than the other LHCI subunits. When Fe stress conditions were continued up to 72 h, the remaining Lhca subunits were lost, with the exception of Lhca6. Each LHCI protein lost from the PSI-LHCI supercomplex results in a loss of 10–14 chlorophyll molecules [38]; this leads to a dramatic decrease in PSI absorption cross-section and is likely to be one of the causes of stress-induced chlorosis.

### SOD activity analysis

**Figure 8:**
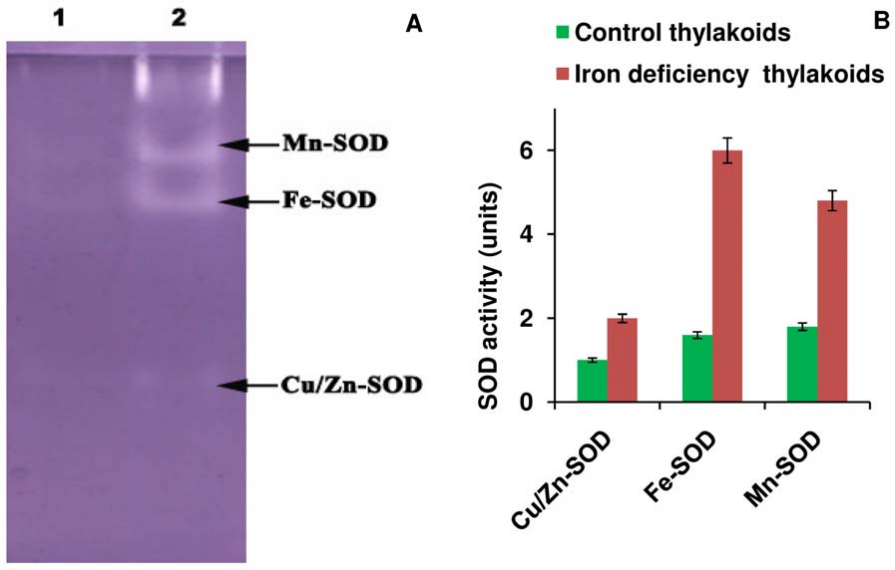
Isoform separation and SOD activity of thylakoid membranes. A) SOD isoforms identified with native staining and B) SOD activity of different isoforms assayed from thylakoid membranes. Lane 1- control thylakoids, and lane 2- thylakoids isolated from cells under Fe deficiency.

Superoxide dismutase enzyme is involved in the environmental stress responses of many species, including both animals and plants [39]. The electrophoretic pattern of three SOD isoforms from thylakoids is shown in Figure 8A. The sensitivity of Cu/Zn-SOD to cyanide (KCN) has been used as a diagnostic tool to distinguish Cu/Zn-SOD from Fe-SOD and Mn-SOD, which are unaffected by cyanide. Likewise, Fe-SOD is irreversibly inactivated by H_2_O_2_, whereas Mn-SOD is resistant to both inhibitors [40]. Among the three isoforms of SOD, Fe-SOD shows more activity in the thylakoid membranes under Fe deficiency (Figure 8B). All SOD forms (Cu/Zn-SOD, Fe-SOD and Mn-SOD) were overexpressed under Fe deficiency condition in thylakoids, indicating that Fe deprivation and oxidative stress are intimately connected in *C. reinhardtii*. These results confirms at the protein level the finding by the proteomics study [15] which showed overexpression of genes involved in the defense against oxidative stress under iron deficiency in *C. reinhardtii*. As PsaC carries the terminal FeS clusters in PSI and PsaD provides the docking site for the electron acceptor ferredoxin, the decrease of PsaC, PsaD and PsaE leads to an impairment of the electron transport chain in PSI. Furthermore, the lack of the stromal subunits hinders docking and electron transfer to ferredoxin which could lead to increased electron transfer from PSI depleted of PsaC, D and E to oxygen. Therefore, loss of the stromal subunits could therefore contribute significantly to the oxidative stress of the cells.

## Discussion

Fe stress conditions clearly impair cell growth in *C. reinhardtii*. The goal of this study has been to establish the degree to which this growth impairment results from changes in the composition of PSI-LHCI supercomplexes. The fluorescence emission results indicate a change in the PSI/PSII ratio during iron deficiency, indicating that PSI is more strongly affected by iron deficiency than PSII (Figure 1C). The 77K emission spectra (Figure 1C and ID), sucrose density gradients (Figure 2), and green gel electrophoresis (Figure 4) give further support to this conclusion (Figure 1C and 1D). The levels of the subunits PsaA and PsaB, which form the core of PSI and bind the majority of the cofactors of the electron transfer chain (ETC) were unchanged (Figure 6).

Major changes were apparent, however, in the membrane-extrinsic subunits PsaC, PsaD, and PsaE, which were already significantly decreased after 72 h of iron deficiency. The effects of iron deficiency on the PSI stromal ridge clearly begin with the loss of PsaE. This seems to be a common feature of stress effects on PSI; complete loss of PsaE was reported in *C. reinhardtii* under high salt conditions [22]. The role of PsaD and PsaE is to stabilize PsaC and the stromal hump which forms the ferredoxin binding site [41], [42]. The loss of PsaC effectively terminates the ETC, as it binds the terminal electron acceptors F_A_ and F_B_ and is essential for the function of PSI [43]. The question arises which factors may contribute to the fast loss of the stromal subunits under iron deficiency. Three factors may contribute to the loss of the stromal hump subunits: 1) inability to *de-novo* synthesize and assemble PsaC, which contains the terminal 4Fe4S clusters FA and FB, due to the lack of iron or 2) controlled degradation of PsaC from intact PSI to use the 12 Fe/PsaC subunit for synthesis of other iron containing proteins in the cell or 3) Unfolding and disassembly of PsaC caused by oxidative damage of the FeS clusters in PSI.

We will first discuss scenario 1: In chloroplasts, Fe-S proteins are present in the electron transfer chain of the photosynthetic apparatus as subunits of both PSI and the Cyt b*6/f* complex. The electron flux from PSI to the dark reactions of photosynthesis is mediated by ferredoxin, another Fe-S protein. Fe-S clusters are therefore essential cofactors for chloroplast functions [44]. In a mutant of Nfu2, a scaffold protein for chloroplast Fe-S sulfur clusters biogenesis, a reduction in PSI activity was observed, resulting from the reduced amount of PSI. The diminished PSI activity may also be attributable to non-functional PSI with compromised 4Fe-4S clusters [45]. According to the *Synechocystis* PSI assembly model [46], assembly is initiated with the integration of the reaction centre subunits (PsaA and B) into membranes. These subunits then acquire their cofactors, including a 4Fe-4S cluster (F_X_) which is coordinated by PsaA and PsaB. This is followed by the docking of PsaC to the stromal surface after the assembly of its two 4Fe-4S centres (F_A_ and F_B_). PsaC does neither fold nor assemble in the absence of the FeS clusters. Thus, a decrease in 4Fe-4S biogenesis on PsaC in the nfu2 mutants can consistently lead to a decrease in functional PSI, which is confirmed by a similar decrease in the level of the PsaD subunit [45]. The successful binding of PsaC to the PsaA/B core is a prerequisite for binding of PsaD and PsaE [47], [48]. Decreased levels of Fe-S clusters in our Fe-stressed cells will thereby inhibit the docking of the stromal ridge proteins PsaC, D and E (Figure 6). The iron limitation will decrease synthesis of Fe-S clusters and may therefore inhibit *de-novo* assembly of the stromal hump of PSI.

This scenario is very plausible, however it cannot play the sole role in the decay of the stromal hump of PSI, as PsaE is lost 24 hours before a significant decay of PsaC is detected. If de-novo synthesis of PsaC would be only contributing factor, the decay of PsaC, PsaD and PsaE should be observed simultaneously. In scenario two, the concerted degradation of subunits of the stromal hump could be triggered by the cells recycling the 12 Fe of PSI for other processes in the cell including enhancement of respiration [16]. When PSI is disassembled *in vitro*, the subunits are lost in the order PsaE, PsaC and PsaD [49] which matches the order of subunits lost under iron depletion for 72 h observed in this work in *C. reinhardtii* and the order of loss of subunits observed under severe prolonged iron depletion for weeks in cyanobacteria [8].

In scenario 3 destruction of the FeS clusters in PSI may be enhanced by oxidative stress. There are reports showing that, in leaves exposed to chilling stress, PSI is affected due to reactive oxygen species generated at the acceptor side [50]. Dismutation of the superoxide anion radicals produces H_2_O_2_, which then reacts with reduced Fe in iron–sulfur centers to form hydroxyl radicals that immediately destroy the iron–sulfur centers. As the redox potential of reduced iron–sulfur centers in PSI is low enough to be able to reduce O_2_, PSI is the main source of ROS such as superoxide anion radicals in plants [50], and a similar effect may be at work under Fe-stress conditions.

The ultrafast fluorescence decay component associated with trapping in the PSI core was virtually unchanged between the control and Fe stress samples (Figure 5A). The 32–34 ps compound arises from the PSI core [31] but there is no difference in fluorescence lifetime or spectral shape between the control and Fe stress samples (Figure 5A) indicating that the PSI core antenna is not affected. Differences were quite apparent in the second component (Figure 5B), which decays on the 120–220 ps timescale. The curves look similar on the blue side of the emission peak, but the stress samples show significantly less emission on the red side than the control sample. Red-shifted pigments in photosynthetic systems typically arise from strongly-coupled dimers of Chl molecules and may exert a significant influence on the exciton trapping dynamics. Previous spectroscopic studies of green algal PSI [35]–[37] have indicated that the PSI core in *C. reinhardtii* does not contain any pigments with transition energies lower than that of P700; longer-wavelength pigments are associated only with the peripheral LHCI complexes. The loss of red-shifted emission in the complexes formed under Fe stress can therefore be attributed to the change in pigment–pigment interactions under Fe stress conditions. This conclusion is also supported by density gradient centrifugation and green gel results, which showed that the largest PSI-LHCI complexes are absent and the LHCI content of the remaining PSI-LHCI complexes are diminished under Fe deficiency (Figure 2 and 4). Similar results have been obtained for *C. reinhardtii* cells grown under salt stress [22], where a decrease on the red side of the LHCI-related decay component was observed. The disruption of the red chlorophyll pigments probably results from a loss of LHCI subunits from the supercomplex and an overall decrease in the quality of PSI-LHCI coupling.

The visible CD data show an increase in amplitude at 675 and 656 nm, which can be attributed to a loss of excitonically coupled Chl *a* in the LHCI antenna. The peak associated with Chl *b* shifts from 641 to 638 nm under Fe deficient conditions (Figure 3), suggesting a change in pigment–pigment interactions that may arise, for example, from a change of the Chl *a*–Chl *b* distance. The green gel results also suggest the loss of LHCI proteins (Figure 4). It was suggested that some of the long wavelength pigments are located at the interface between different LHCI proteins in the complex, which would explain why they are the first to be lost when supercomplex assembly is impaired. The CD data also support the conclusion that LHCI complexes were lost under Fe deficiency conditions. Alternatively, the changes in CD signals of pigments could arise from the modification of interactions between pigment molecules. This data is consistent with other data supported by Figure 1A, 3, 4 and 6.

Oxidative stress may also play a role in the effects of Fe deprivation on *C. reinhardtii*. Oxidative stress in many species involves the expression of different SOD isoforms having different molecular weights [51], [52]; in general, Fe-SOD is more highly expressed in the chloroplast than other SOD isoforms [51], [53]. Fe stress is associated with an increase in SOD activity; presumably to cope with the higher levels of reactive oxygen species (ROS) generated under Fe stress. Similar results have been observed in *Arabidopsis* under cadmium and copper stress [54], in winter wheat leaves under the effect of exogenous hydrogen peroxide [55], and in sacred lotus under chilling and oxidative stresses [56].

Paradoxically, a shortage of cellular Fe could lead the remaining iron-sulfur clusters to be increasingly dangerous to the already-impaired cell. The loss of PsaC, PsaD, and PsaE at the reducing side of PSI will terminate the electron transfer chain and may cause accumulation of electrons at F_X_, which is fully exposed to solvent when PsaC, PsaD, and PsaE are lacking. These results agree with a report of a deletion mutant of *psaE* in the cyanobacterium *Synechocystis sp.* strain PCC 6803, which shows increased photodamage due to the formation of oxygen radicals [57].

Our immunoblot studies of the LHCI subunits reveal the order of events that lead to a decrease of the individual LHCI subunits under Fe stress conditions. Interestingly, the different LHCI proteins show very significant differences in their sensitivity to Fe stress. In higher plants such as pea, four LHC proteins (Lhca1 to 4) surround the PSI core in a half-moon arrangement [34]. In *C. reinhardtii*, on the other hand, a large complex is formed in which a second layer of 10–12 LHCI proteins is attached to this core PSI-LHCI complex. The LHCI proteins can be arranged in order of their susceptibility to loss under Fe stress: Lhca1, Lhca9, Lhca2, Lhca7, Lhca8, Lhca3, Lhca4 and Lhca5 (Figure 7). Lhca6 is unique; it was actually more stably bound to PSI after 72 h of Fe deficiency than in the control. Our group recently reported a 3D model in which Lhca6 is embedded close to the PSI core while Lhca1 and 9 form the outer shell of the larger PSI-LHCI supercomplex [58].

The decay of PSI may be a very well orchestrated event which starts with the decoupling of the LHCI complexes. As discussed above, the lack of the FeS clusters and impairment of the stromal hump increases oxidative stress upon electron transfer from PSI to oxygen, which can be minimized by decoupling of the antenna, before the electron transport chain is hampered by the lack of PsaC.

One important question that has to be addressed is why the stress response differs so much between green algae and cyanobacteria. In cyanobacteria, Fe stress leads to the expression of the IsiA protein which forms double rings surrounding one PSI trimer, which contains up to 43 IsiA antenna proteins attached to the PSI trimer, increasing the absorption cross section of PSI by a factor of three and maintaining electron flow through PSI [8]. This adaptation allows the cyanobacterial cells to grow under Fe deficiency in nanomolar Fe concentration without significant chlorosis for weeks. Only after Fe is completely absent for more than a month is the loss of PsaC, PsaD and PsaE detected in *T. elongatus* [8], after which severe chlorosis sets in. *C. reinhardtii*, on the other hand, undergoes severe chlorosis after only 72 h of Fe deficiency (Figure 1A) and is thereby much more prone to Fe stress than cyanobacteria. Instead of increasing the antenna size of PSI, the LHCI proteins in green algae are removed from the PSI core, decreasing the absorption cross section of PSI. The question arise why cyanobacteria compensate in part for the lower amount of PSI by increasing its light harvesting cross section, while the antenna size of PSI is decreased in *C. reinhardtii*.

The most likely reason for this counter-intuitive behaviour is the lower stability of the stromal hump in green algae compared to cyanobacteria. As described above, PSI complexes that have been affected by stress may actually exacerbate the situation by generating additional ROS. Under such conditions, the loss of LHCI proteins may in fact be advantageous – the cell's best survival strategy could be to reduce the rate of electron flow through PSI by reducing its antenna size. An important related question is why some cyanobacteria can maintain an intact PSI for weeks while the ETC in *C. reinhardtii* is affected after only 72 h. *C. reinhardtii* has evolved to grow in fresh water under Fe-replete conditions, and has not developed Fe acquisition and storage mechanisms as efficient as those in cyanobacteria [59]. Cyanobacteria, on the other hand, have a very efficient Fe transport and chelating system that allows them to access Fe even from stainless steel parts in bioreactors, so that Fe deficiency can only be induced in pure glass and plastic vessels. In addition, many cyanobacteria store large amounts of Fe inside the cell so that the intracellular Fe level sinks to nanomolar concentrations only after three weeks of growth in medium without Fe. This allows the cells to maintain an intact PSI and ETC for a prolonged time, during which of the formation of the IsiA ring increases the efficiency of PSI. In addition, *C. reinhardtii* is a non-obligate photoautotroph, *i.e.*, it can also grow non-photosynthetically using various carbon sources. In contrast, cyanobacteria are obligate photoautotrophs and lack this metabolic flexibility. An active electron transport chain with both PSI and II is therefore absolutely essential for the survival of cyanobacterial cells. This has created greater pressure for the evolution of an adaptation mechanism that allows cells to maintain their photosynthetic apparatus under stress conditions. *C. reinhardtii*, however, can grow photoautotrophically and heterotrophically and therefore does not depend solely on photosynthesis for survival. It has therefore developed an alternate strategy for survival where iron from disassembled PSI may be used synthesize other protein complexes including Fe-superoxide dismutase and protein complexes involved in respiration. This hypothesis agrees very well with the finding of Naumann *et al* [16] who detected an increase of respiratory activity and increase in subunits of all the 4 complexes of the respiratory chain and the ATP-synthase under iron deficiency in *C. reinhardtii*.

In summary, Fe deficiency leads to a rapid decrease in the levels of photosynthetic pigments and a substantial decrease in photochemical electron transport. The acceptor side proteins of PSI are strongly affected by Fe stress, thereby interrupting electron transfer. The assembly of LHCI subunits and their incorporation into PSI-LHCI supercomplexes are hampered under iron deficiency as chlorophyll synthesis requires Fe. These results show that different oxygenic photosynthetic organisms have developed very different strategies for survival under stress conditions, depending on their ecological niche and their ability to adjust their metabolism in response to environmental stresses. Oxygenic photosynthetic organisms have successfully colonized a dizzying variety of habitats, from deserts to deep oceanic waters, and appropriate stress responses may have played an important role in this diversification.
